# Comprehensive Analysis of the Global Protein Changes That Occur During Salivary Gland Degeneration in Female Ixodid Ticks *Haemaphysalis longicornis*

**DOI:** 10.3389/fphys.2018.01943

**Published:** 2019-01-22

**Authors:** Hui Wang, Xiaoli Zhang, Xiao Wang, Baowen Zhang, Minjing Wang, Xiaolong Yang, Xuying Han, Rui Wang, Shuguang Ren, Yuhong Hu, Jingze Liu

**Affiliations:** ^1^Key Laboratory of Animal Physiology, Biochemistry and Molecular Biology of Hebei Province, College of Life Sciences, Hebei Normal University, Shijiazhuang, China; ^2^The Fourth Hospital of Hebei Medical University, Shijiazhuang, China; ^3^Instrumental Analysis Center, Hebei Normal University, Shijiazhuang, China

**Keywords:** tick, salivary gland, apoptosis, iTRAQ, RNA interference (RNAi)

## Abstract

Ticks are notorious blood-sucking arthropods that can spread a variety of pathogens and cause great harm to the health of humans, wildlife and domestic animals. The salivary glands of female ticks degenerate rapidly when the ticks reach critical weight or become engorged, which can be caused by hormones and by the synergistic effects of multiple proteins. To explore the complex molecular mechanisms of salivary gland degeneration in ticks, this study applies iTRAQ quantitative proteomic technology for the first time to study changes in protein expression in the salivary glands of female *Haemaphysalis longicornis* during the process of degeneration and to search for proteins that play an important role in salivary gland degeneration. It was found that the expression of some proteins associated with energy production was continuously down-regulated during salivary gland degeneration, while some proteins associated with DNA or protein degradation were consistently up-regulated. Furthermore, the expression of some proteins related to cell apoptosis or autophagy was also changed. These proteins were knocked down by RNAi to observe the phenotypic and physiological changes in female ticks. The results showed that the time required for engorgement and the mortality rates of the female ticks increased after RNAi of F0F1-type ATP synthase, NADH-ubiquinone oxidoreductase, cytochrome C, or apoptosis-inducing factor (AIF). The corresponding engorged weights, oviposition amounts, and egg hatching rates of the female ticks decreased after RNAi. Interference of the expression of AIF in engorged ticks by RNAi showed that the degeneration of salivary glands of female ticks was slowed down.

## Introduction

Ticks are special blood-sucking parasitic arthropods that can transmit a variety of pathogens, including viruses, bacteria, protozoa, and nematodes. Examples of these pathogens include tick-borne encephalitis virus (Selinger et al., 2017), *Borrelia burgdorferi* (causing Lyme disease) (Marchant et al., 2017), *Rickettsia* (causing tick-borne rickettsiosis) (Kartashov et al., 2017), *Anaplasma phagocytophilum* (causing human granulocytic anaplasmosis) (Schotthoefer et al., 2018), *Babesia* (Abdullah et al., 2018), and filarial nematodes (Zhang et al., 2011), which cause various diseases in humans and animals (Estrada-Peña et al., 2012). But at present, research on ticks is obviously lagging behind. Multifaceted research on ticks need to be carried out urgently.

The species *Haemaphysalis longicornis* belong to the genus *Haemaphysalis*, which is a member of the Ixodidae family. *H. longicornis* is widely distributed in some East Asian countries and Australia (Hoogstraal et al., 1968). This species is one of the most harmful ticks in the world. Therefore, it is of great importance to carry out comprehensive studies of this species.

The salivary glands of ticks secrete large amounts of saliva into the host when the ticks are sucking blood (Šimo et al., 2017). Tick saliva contains a variety of enzymes and peptides that inhibit host blood coagulation, tissue repair, inflammatory reactions, and immune responses (Tirloni et al., 2015), allowing ticks to suck blood uninhibitedly. Therefore, salivary gland development is of great significance for ticks. It takes approximately 5–10 days for female ticks to start sucking blood to become engorged (Ullah and Kaufman, 2014), and the salivary glands develop rapidly during this period, but when the female ticks become engorged, their salivary glands begin to degenerate (Friesen and Kaufman, 2002). Ecdysteroid hormones can control the degeneration of salivary glands (Harris and Kaufman, 1985; Ullah and Kaufman, 2014). In addition, some proteins, such as caspases, may also play an important role in the degeneration of salivary glands (Yu et al., 2017). To explore whether there are other proteins that can promote salivary gland degeneration and analyze the global changes in protein levels that occur during the process of salivary gland degeneration, a quantitative proteomic analysis was carried out in this study. To the best of our knowledge, this study is the first to use an iTRAQ (isobaric tags for relative and absolute quantification)-based quantitative proteomic approach to examine the dynamic changes in the levels of all proteins during salivary gland degeneration in female *H. longicornis*.

Because the genome and transcriptome sequences of *H. longicornis* are not available in GenBank, we first carried out transcriptome sequencing of female *H. longicornis* to build a database. After protein function annotation, this database was used for subsequent quantitative proteomic studies to analyze the mechanism of salivary gland degeneration at the protein level. About 8,800 ticks were dissected for proteomic studies. As a result, a total of 3419 high confidence proteins were identified from the salivary glands of the engorged female ticks from 0 to 72 h after detachment from the host. In addition, 225 proteins were continuously up-regulated and 476 proteins were continuously down-regulated during this stage. Through bioinformatics analysis, we identified some proteins that were associated with salivary gland degeneration and marker proteins that were produced during rapid salivary gland degeneration. Some proteins that play an important role during salivary gland degeneration can severely affect salivary gland structure and function in ticks and can even affect the development of tick bodies. We selected some of the proteins and then used RNAi to interference the selected proteins one by one. The results showed that after interference of the proteins F0F1-type ATP synthase, NADH-ubiquinone oxidoreductase, cytochrome C, and AIF, female *H. longicornis* were unable to suck blood normally and became prematurely detached from the host body or even sterile. In the meantime, interference of AIF and lysosomal acid phosphatase by RNAi showed that the degeneration of salivary glands of female ticks was slowed down, suggesting that AIF can promote apoptosis of tick salivary gland cells. This study of these genes in ticks provides new targets for the screening of anti-tick drugs.

## Materials and Methods

### Tick Feeding

*Haemaphysalis longicornis* were collected from Zhangjiakou in Hebei Province, China. The ticks were allowed to feed on the ears of New Zealand white rabbits. When not feeding, the ticks were cultured at 25 ± 1°C and 75% relative humidity in artificial climate incubators. All experimental procedures were approved by the Animal Ethics Committee of Hebei Normal University (protocol number: IACUC-157026).

### mRNA Extraction and cDNA Library Construction

Approximately 300 female *H. longicornis* at different feeding stages were prepared for RNA extraction. Total RNA was extracted from whole ticks (after removal of host blood from their midguts) using TRIzol Reagent (Invitrogen, United States). RNA purity and concentration were tested by using a Qubit 2.0 (Invitrogen, United States) and Bioanalyzer 2100 (Agilent Technologies, United States). mRNA was purified from the total RNA using an mRNA isolation kit (Qiagen, Germany) according to the manufacturer’s protocol. The NEBNext^®^ Ultra^TM^ RNA Library Prep Kit for Illumina^®^ (NEB, United States) was used according to the manufacturer’s protocol to construct cDNA libraries. Illumina’s proprietary fragmentation buffer was added to randomly disrupt the mRNA and obtain 200 bp (±25 bp) fragments. Random primers, dNTPs, and ProtoScript^®^ II reverse transcriptase were used for first-strand cDNA synthesis. Second-strand cDNA synthesis was performed using the NEBNext second-strand synthesis enzyme mix. After using oligo dT magnetic beads to purify the cDNA, the cDNA was attached to the Illumina PE adapter, and the cDNA libraries were finally constructed. Clustering of the index-coded samples was performed on a cBot cluster generation system using the TruSeq PE Cluster Kit v3-cBot-HS (Illumina, United States) according to the manufacturer’s instructions.

### Illumina Sequencing, Assembly and Annotation

Transcriptome sequencing was performed from both the 5′ and 3′ paired-ends on an Illumina HiSeq 2500 sequencer at Biomarker Technologies (Beijing, China). The single-ended sequencing read length was PE125. Sequencing reads were obtained after image analysis and base calling. Adaptor sequences were trimmed, and low-quality reads, which contained more than 5% ambiguous ‘N’ nucleotides or more than 50% bases with quality scores lower than 5, were removed. The Q20, Q30 and sequence duplication levels were calculated based on the clean reads. Then, these high-quality RNA-Seq reads were assembled into contigs using Trinity software (Grabherr et al., 2011), and the parameters were set to default values. Subsequently, these contigs were clustered, and components were obtained (there exists an overlap of *K*-1 bases between two adjacent contigs, and a certain number of *k*-mers are required, half of which are compared to the two contigs; such contigs converge to components). De Bruijn graphs were constructed for contigs in each component; the resulting De Bruijn graphs were simplified; and the De Bruijn diagram was untied with real reads. Finally, the longest transcripts were obtained as unigenes, and the redundancies were filtered. Open reading frames (ORFs) were predicted using the Getorf program of the EMBOSS package (version 6.3.1). CDSs (coding sequences) were predicted using Trinity software. Then, the CDSs were translated into protein sequences and functional annotation was performed by searching against the arthropod Nr (non-redundant protein sequences; February 6, 2015) database of the National Center for Biotechnology Information (NCBI) using the BLAST algorithm with a cut-off E-value of 10^-5^ and the HMMER version 3.0 software package with a cut-off *E*-value of 10^-10^. The ORF sequences without annotations and CDSs with annotations were combined, and these protein sequences were saved as fasta format files, which were used as a database for subsequent quantitative proteomic studies.

### Tissue Dissection and Protein Extraction

The time of natural detachment of engorged females from the host was recorded as 0 h. Salivary glands from female ticks at the 0, 24, 48, or 72 h post-engorgement stages were dissected. Then, the salivary glands were immediately placed in sterile PBS (1 M) containing a protease inhibitor cocktail (Roche, Mannheim, Germany) and rapidly frozen at -80°C for subsequent experiments. Salivary glands from approximately 550 engorged females from each processing group (in all, about 6,600 ticks) were collected in a pre-cooled glass homogenizer and ground in PBS (1 M) containing the protease inhibitor cocktail (Roche, Mannheim, Germany). Then, the homogenate was transferred into a 50 ml centrifuge tube and centrifuged for 20 min (4°C, 12,000 ×*g*), and the supernatant was transferred to a new 50 ml centrifuge tube. Then, 10 ml of Tris-saturated phenol (pH 7.8) was added, and the mixture was vortexed for 5 min and centrifuged for 20 min (4°C, 12,000 ×*g*). Subsequently, 10 ml of 50 mM Tris-HCl (pH 8.0) was added, and the mixture was vortexed for 5 min and centrifuged for 20 min (4°C, 12,000 ×*g*). After removing the upper aqueous phase, the protein content was precipitated by adding a certain volume of 0.1 M ammonium acetate in methanol and storing at -20°C overnight. The mixture was centrifuged for 20 min (4°C, 12,000 ×*g*), and the supernatant was discarded. The protein pellets at the bottom of the tube were further washed with methanol, and the extracted proteins were lyophilized.

### Protein Digestion

Three hundred micrograms of protein sample from each processing group was reduced with 10 mM dithiothreitol by incubating for 30 min at 37°C. Then, the proteins were alkylated with 20 mM iodoacetamide at 25°C in the dark with constant shaking for 45 min to stabilize the sulfhydryl groups. The samples were then washed and digested using filter-aided sample preparation (FASP) with an ultrafiltration centrifuge tube that had a 10-kDa molecular weight cut-off (Wiśniewski et al., 2009). Both protein washing and enzyme solution preparation were performed using the dissolution buffer (pH 8.0) provided in the iTRAQ Kit (AB Sciex, United States). The enzymatic reaction with trypsin (1:20 w/w, Promega, United States) was strictly controlled at 37°C for 12 h. After the final enzyme treatment, the peptides were eluted from the ultrafiltration membrane with the dissolution buffer (by centrifuging at 12,000 ×*g*), and the filtrate that collected in the bottom of the tube was used for subsequent iTRAQ-based proteomic analysis. The purified peptide concentrations were then normalized to a common value using a BCA Protein Assay Kit (Pierce Biotechnology) and LC-MS (Thermo Fisher Scientific, United States). Enzyme efficiency was monitored by LC-MS.

### iTRAQ Labeling

**FIGURE 1:**
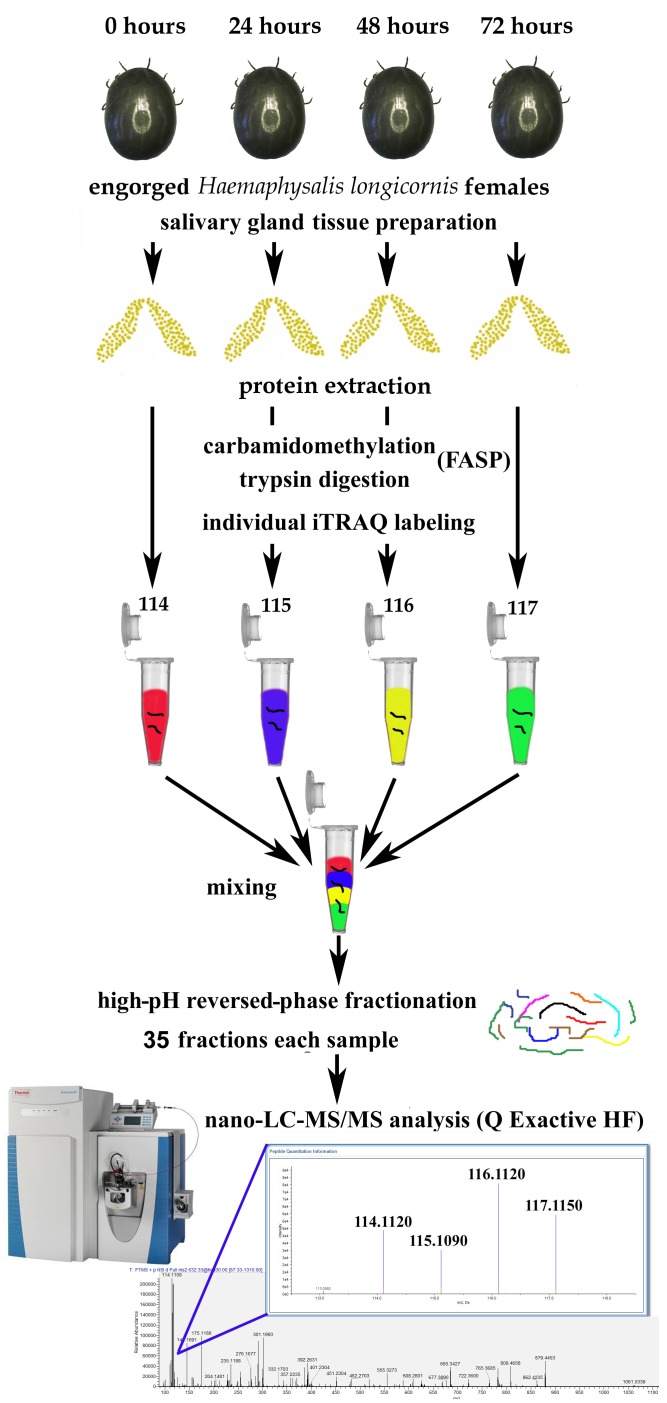
Workflow for iTRAQ-based analysis of global protein expression during salivary gland degeneration in female *Haemaphysalis longicornis.*

The workflow for iTRAQ labeling is shown in Figure 1. Each sample was labeled with the 4-plex iTRAQ reagents (114, 115, 116, and 117) for 3 h according to the manufacturer’s instructions. After labeling, twice the volume of deionized water was added to all the samples, which were then hydrolysed for 15 min to terminate the reaction. Then, the four samples were mixed together.

### LC-MS Analysis

Eight hundred micrograms of the iTRAQ-labeled mixed peptide samples was separated by high-pH C18 reversed phase high performance liquid chromatography (RP-HPLC) (Agela; 5 μm particle size, 100 Å pore size, 0.46 cm × 25 cm). After sample loading, the concentration of acetonitrile (ACN) (containing 5 mM ammonium formate, pH 10.0) in the eluting solvent was increased to 45% (v/v) over 90 min at a flow rate of 1 ml/min using a linear gradient. Thirty-five fractions were analyzed by LC-MS using a nanoACQUITY UPLC M-Class system (Waters, United States) and Q Exactive HF mass spectrometer (Thermo Fisher Scientific, United States). Each sample was first loaded onto a C18 RP trap column (5 μm particle size, 100 Å pore size, 180 μm ID × 20 mm length; Waters, United States) and then separated on a C18 RP analytical column (1.8 μm particle size, 100 μm ID × 150 mm length; Waters, United States) at a flow rate of 300 nL/min using a linear ACN gradient 0 ∼ 40% solvent B over 75 min (solvent A: 99.9% H_2_O, 0.1% formic acid; solvent B: 99.9% ACN, 0.1% formic acid). The eluted peptides were electrosprayed into the mass spectrometer with a 2.5 kV electrospray voltage. LC-MS data were acquired in data-dependent acquisition mode. Full scans were acquired in a range of 350–1800 *m*/*z* with an isolation width of 1.5 *m*/*z* at a resolution of 70,000. MS/MS spectra of the 10 most intense ions per cycle were fragmented with high-energy collisional dissociation (HCD) at a resolution of 17,500. The dynamic exclusion was set to 20 s. In addition, the maximum ion injection time was set to 250 ms, and the automatic gain control (AGC) target value was set to 1 × 10^6^ ions. Three test replicates were performed for iTRAQ analysis.

### Protein Identification and iTRAQ Quantification

The MS/MS data for 35 fractions from each test were combined and then analyzed by Proteome Discoverer (version 2.2) software (Thermo Fisher Scientific, United States) using the SEQUEST algorithm. The database derived from transcriptome sequencing of *H. longicornis* consisted of 498748 sequences. The following search parameters were applied: (1) precursor mass tolerance,10 ppm; (2) fragment mass tolerance, 0.02 Da; (3) enzyme, trypsin (2 missed cleavages allowed); (4) fixed modifications, iTRAQ 4-plex labels (N-terminal and lysine residues, 144.102 Da) and carbamidomethylation of cysteine residues (57.021 Da); (5) variable modifications, methionine oxidation (15.995 Da); (6) activation type, HCD; (7) the “Total peptide amount” tab was checked to normalize proteins by the protein ratio median; and (8) to estimate the false discovery rate (FDR), a decoy database search was performed simultaneously. *Q*-value was used as the criterion to evaluate the FDR, and the target FDR (strict) was set to 0.01. Only proteins quantified with at least two unique peptides and “high” confidence (FDR < 1%) were considered for further analysis. All protein change ratios were transformed to base 2 logarithm values. In the base 2 logarithm space, a 1.5-fold change in levels is reported as -0.585 and 0.585 for down-regulation and up-regulation, respectively.

### Bioinformatics Analysis of Differentially Expressed Proteins

To further reveal the biological functions of these differentially expressed proteins, some bioinformatics methods were used. The open source software GProX was used for cluster analysis of differentially expressed proteins with similar change patterns (Rigbolt et al., 2011). The number of clusters was set to 4, and a fixed regulation threshold (upper limit of 0.58 and lower limit of -0.58, corresponding to the original ratios of approximately 1.5 and 0.67) was used. The minimal membership for the plot was set as 0.5. Other parameters were set to default values. The Gene Ontology (GO) functional enrichment of the differentially expressed proteins was analyzed using the PANTHER classification system^1^. Pathways associated with the differentially expressed proteins were identified using the Kyoto Encyclopedia of Genes and Genomes (KEGG) database^2^. The protein–protein interactions (PPIs) were explored using the Search Tool for the Retrieval of Interacting Genes/Proteins (STRING) online program (version 10.5^3^). The Multiple proteins category was selected, and the differentially expressed proteins were entered; *Ixodes scapularis* was selected as the species. The online system parameters were set as follows: (1) the meaning of network edges: evidence; (2) active interaction sources: experiments and databases; (3) network display mode: interactive; (4) display simplifications: hide disconnected nodes in the network; and (5) other parameters: default. Heat map analysis was conducted using Cluster 3.0 software.

### RNA Interference

Double-stranded RNA (dsRNA) was synthesized for interference using the RNAi Kit (Promega, United States), as described by de la Fuente et al. (2007). The targeting positions 683–1028, 16–323, 64–351, 361–768, and 1015–1312 of the F0F1-type ATP synthase, NADH-ubiquinone oxidoreductase, cytochrome C, AIF, and lysosomal acid phosphatase nucleotide sequences, respectively, were proven to be specific by searching the transcriptome sequence of *H. longicornis*. After the target mRNA was cloned and sequenced, the correct cDNA sequences were used for subsequent dsRNA synthesis. The sequences of the primers used to synthesize the dsRNA contained the T7 promoter sequence (underlined): 5′-TAATACGACTCACTATAGGGAGGCAAGATTGGGCTGTT-3′ and 5′-TAATACGACTCACTATAGGGCCTGGGTGAACCTGAAAA-3′ (F0F1-type ATP synthase); 5′-TAATACGACTCACTATAGG GTGAAGTCTACAGCACGAAC-3′ and 5′-TAATACGACTCACTATAGGTCGGGTTGACAGTTTCC-3′ (NADH-ubiquinone oxidoreductase); 5′-TAATACGACTCACTATAGG GTGGGTCACCTTCCTTGC-3′ and 5′-TAATACGACTCACTATAGGAGATGAACAGCGGTCC TTG-3′ (cytochrome C);5′-TAATACGACTCACTATAGGACCGTCTACCAGGTGTTCT CC-3′ and 5′-TAATACGACTCACTATAGGCTCGGCGTTGGC TTTGA-3′ (AIF); and 5′-TAATACGACTCACTATAGGTGTGG AACAGCGAGGTGG-3′ and 5′-TAATACGACTCACTATAGGG GTCAGAGTGCCGCA AAA-3′ (lysosomal acid phosphatase). Green fluorescent protein (accession number KX247384.1) dsRNA was synthesized as the control at the same time. Twenty unfed female *H. longicornis* were injected with 0.5 ∼ 1 μl (approximately 4 μg/μl) of dsRNA using a Hamilton syringe (33-gauge needle) in the lower right quadrant of the body as described previously (Kocan et al., 2009). Then, these injected ticks were cultured at 25 ± 1°C and 75% relative humidity in artificial climate incubators for 1 day for recovery, after which the ticks were allowed to infect New Zealand rabbits. When applying RNAi on engorged ticks, the ticks were injected with dsRNA at the time point when they fall off the hosts. The degree of knock down was confirmed by test mRNA expression levels using qRT-PCR. Digital Microscope (DVM6, Leica, Germany) was used to observe the changes of salivary glands in ticks. The effect of RNAi was evaluated by measuring the following parameters: (1) tick mortality rate; (2) time required for engorgement and weight of engorged tick; (3) the oviposition amount; and (4) egg hatching rate.

## Results

### Sequencing Results and Gene Annotation

To use the iTRAQ quantitative proteomic method to globally analyze the dynamic changes in protein levels during the process of rapid degeneration of *H. longicornis* salivary glands, we needed to first establish a protein sequence database for female *H. longicornis*. Therefore, we used an Illumina HiSeq 2500 sequencer to measure the transcript levels of female *H. longicornis* and predicted CDSs. Then, the CDSs were translated into protein sequences, and functional annotation was performed. These protein sequences were eventually used as a database for subsequent iTRAQ proteomic studies. The base percentage of the database reached 93.08% (≥Q30, with an error probability of 0.001). Sequencing results showed that a total of 28,468,245 clean reads were generated. The number of mapped paired-end reads was 23,616,159, and the mapped proportion was 82.96%. The assembly received 121,855 transcripts (N_50_ = 1,716) and 87,825 unigenes (*N*_50_ = 1,140). Among these unigenes, 13,222 were longer than 1 kb. The length statistics of the assembled contigs, transcripts, and unigenes are shown in Supplementary Table S1. The FPKM (fragments per kilobase of transcript per million mapped reads) values of these genes are displayed in Supplementary Figure S1. The statistics for the length distributions of all the unigenes are shown in Supplementary Figure S2. The ORFs were predicted using the Getorf program, and gene expression was simulated using the BLAST algorithm method. The ORF sequences without annotations and the CDSs with annotations were combined, and these protein sequences were saved as fasta format files, which were used as a database for subsequent proteomic studies.

### Overview of the Quantitative Proteomic Experimental Design

After some female ticks become engorged and detach from the host, their salivary glands begin to degenerate rapidly (Nunes et al., 2016). The morphological changes during this period are mainly manifested as a decrease in the number of acini, nuclear breakdown, and cell fragmentation (Scopinho et al., 2008). However, what are the dynamic changes in protein levels that occur in the salivary gland during degeneration, and how does the coordination of these proteins eventually cause complete degeneration of the salivary glands? This study was designed to answer these questions. In this experiment, the total protein samples extracted from the degenerated *H. longicornis* salivary glands at different post-engorgement time points were labeled with the iTRAQ reagents, and LC-MS/MS was used for data acquisition (Figure 1). Then, a data search was carried out using Proteome Discoverer (version 2.1) software. Finally, the identification and quantitative results for the proteins were obtained.

### Protein Identification

**FIGURE 2:**
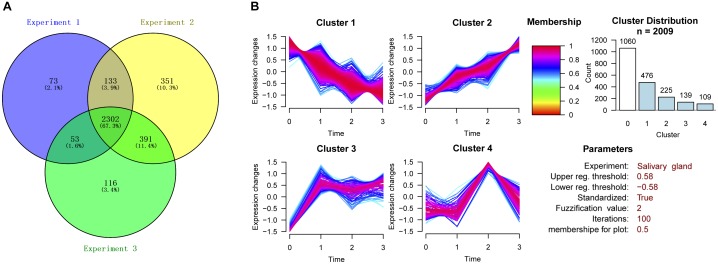
Cluster analysis of differentially expressed proteins in the three experiments. **(A)** Venn diagram showing the number of proteins identified in degenerated *H. longicornis* salivary glands among the three replicate experiments. **(B)** Cluster analysis of differentially expressed proteins.

The three experiments identified 3952, 4714, and 4298 proteins. Only proteins quantified with at least 2 unique peptides and “high” confidence (FDR < 1%) were considered. Therefore, after filtration, the number of identified proteins decreased to 2561, 3177, and 2862, respectively. A total of 3419 high confidence proteins were identified. The number of proteins identified in all three experiments was 2302, accounting for 67.3% of the total proteins (Figure 2A). Subsequent data analysis was conducted on the basis of these 2302 proteins (Supplementary Table S2).

### Cluster Analysis of Differentially Expressed Proteins

The proteins with CV ≤ 30% in the quantitative results from the three experiments were selected for further cluster analysis. The proteins with change ratios ≥ 20 or ≤0.05 were not considered for the analysis. The construction of clusters was based on all the quantified proteins from the 115:114, 116:114, and 117:114 groups. We divided these proteins into four clusters according to the trend for the change in protein expression in the four post-engorgement stages (Figure 2B). Proteins with values higher than 1.5 or lower than 0.67 were considered to be up-regulated or down-regulated (corresponding to log 2 values of 0.58 and -0.58), respectively.

As a result, 1060 proteins exhibited no significant change in expression (Cluster 0). The expression patterns of the up-regulated or down-regulated proteins were generally divided into four types by GProX clustering analysis: Cluster 1 contained 476 proteins, Cluster 2 contained 225 proteins, Cluster 3 contained 139 proteins and Cluster 4 contained 109 proteins.

The overall change trend for the expression of the 476 proteins in Cluster 1 from the day of female engorgement (0 h) to the 3rd day post engorgement (72 h) was continuous down-regulation. The proteins in Cluster 1 were mainly enzymes, such as transferases, oxidoreductases, hydrolases, and isomerases; in addition, this cluster also contained nucleic-acid-binding proteins, enzyme modulators, and chaperones. In contrast, the overall change trend for the expression of the 225 proteins in Cluster 2 from 0 to 72 h was up-regulation. The most common protein type in Cluster 2 was nucleic-acid-binding proteins, followed by some hydrolases and enzyme modulators. The expression of 139 proteins in Cluster 3 exhibited a trend that consisted on initial up-regulation (0–24 h), followed by a constant expression level. Most proteins in Cluster 3 were also nucleic-acid-binding proteins, followed by enzyme modulators and transporters. The changes in expression of the 109 proteins in Cluster 4 were irregular. The most abundant proteins in Cluster 4 were hydrolases, followed by transferases and oxidoreductases.

### GO Function Annotation of Differentially Expressed Proteins

The GO functions were annotated for all the differentially expressed proteins in the following groups: 24 h post engorge-ment:engorgement (0 h) (115:114); 48 h post engorgement:engorgement (0 h) (116:114); and 72 h post engorgement:engorgement (0 h) (117:114). As shown in Supplementary Figure S3, these proteins were all grouped into three major functional groups: molecular function, biological process, and cellular component. Further enrichment analysis was conducted in these three categories. A total of 8 terms were enriched in the molecular function category. Among these terms, the catalytic activity and binding terms accounted for the largest proportion of proteins. There were a total of 12 terms in the biological process category, among which the metabolic process and cellular process terms contained the largest proportion of proteins. There were a total of six terms in the cellular component category, among which, cell part accounted for the largest proportion of proteins.

**FIGURE 3:**
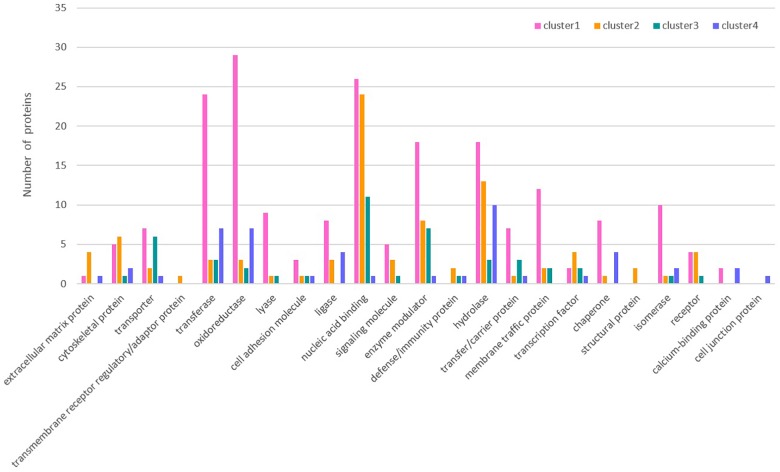
Protein classification based on GO functional annotations of each cluster protein.

The classification of each cluster protein based on its GO functional annotations using the PANTHER classification method^4^ is shown in Figure 3. Simultaneously, GO annotation was also performed for the differentially expressed proteins in Clusters 1 ∼ 4 (Supplementary Figures S4–S7). In Cluster 1, the proteins assigned to the molecular function category were grouped into eight terms. Among these terms, catalytic activity (51.8%) and binding (26.2%) accounted for the largest proportion. The proteins that were grouped into the catalytic activity term were further annotated in detail and divided into seven terms, namely, transferase activity, enzyme regulator activity, hydrolase activity, oxidoreductase activity, lyase activity, ligase activity, and isomerase activity. The proteins assigned to the biological process category were grouped into 10 terms. Among these terms, metabolic process (39.1%) and cellular process (30.0%) accounted for the largest proportion. The proteins that were grouped into the metabolic process term were further annotated in detail and divided into 9 terms. Among these terms, primary metabolic process (16.9%) accounted for the largest proportion. The proteins assigned to the cellular component category were grouped into eight terms. Among these terms, cell part (50.3%) accounted for the largest proportion. The proteins that were grouped into the organelle term were further annotated in detail and divided into seven terms.

In Cluster 2, the proteins assigned to the molecular function category were grouped into seven terms. Among these terms, catalytic activity (41.8%) and binding (34.2%) accounted for the largest proportion. The proteins that were grouped into catalytic activity were further annotated in detail and divided into seven terms, namely, transferase activity, enzyme regulator activity, hydrolase activity, oxidoreductase activity, lyase activity, helicase activity, and ligase activity. The proteins assigned to the biological process category were grouped into 12 terms. Among these terms, metabolic process (32.1%) and cellular process (30.3%) accounted for the largest proportion. The proteins that were grouped into the metabolic process term were further annotated in detail and divided into seven terms. Among these terms, primary metabolic process (15.2%) accounted for the largest proportion. The proteins assigned to the cellular component category were

grouped into six terms. Among these terms, cell part (39.6%) and organelle (30.2%) accounted for the largest proportion. The proteins that were grouped into the organelle term were further annotated in detail and divided into six terms.

Simultaneously, GO function annotation was also carried out for differentially expressed proteins in different post-engorgement stages. That is, as shown in Supplementary Figure S8, the differentially expressed proteins from the following groups were subjected to GO annotation: 24 h post engorgement:engorgement (0 h) (115:114); 48 h post engorgement:24 h post engorgement (116:115); 72 h post engorgement:48 h post engorgement (117:116).

There were 391 differentially expressed proteins in the salivary gland in the 24 h post engorgement:engorgement (0 h) (115:114) group; among these proteins, 203 were up-regulated and 189 were down-regulated. There were 292 differentially expressed proteins in the salivary gland in the 48 h post engorgement:24 h post engorgement (116:115) group; among these protein, 152 were up-regulated and 140 were down-regulated. There were 177 differentially expressed proteins in the salivary gland in the 72 h post engorgement:48 h post engorgement (117:116) group; among these proteins, 55 were up-regulated and 122 were down-regulated.

### KEGG Pathway Analysis of Differentially Expressed Proteins

**FIGURE 4:**
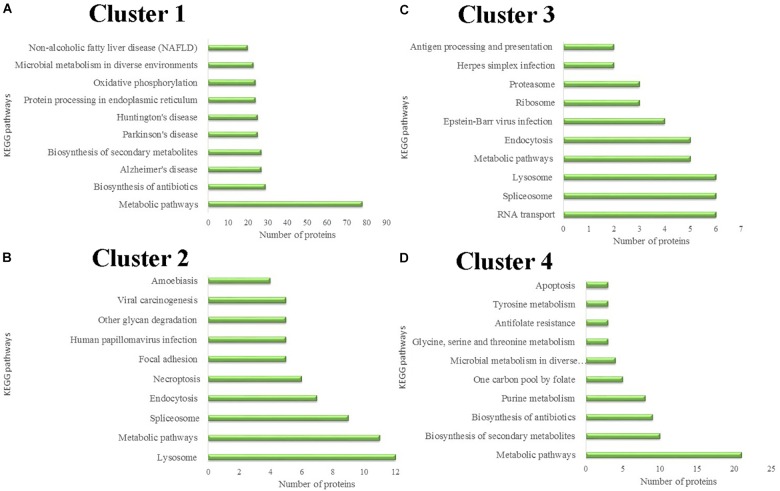
Protein classification based on KEGG enrichment analysis of differentially expressed proteins in each cluster. **(A)** KEGG pathways analysis of proteins in cluster 1. **(B)** KEGG pathways analysis of proteins in cluster 2. **(C)** KEGG pathways analysis of proteins in cluster 3. **(D)** KEGG pathways analysis of proteins in cluster 4.

Differentially expressed proteins in the four different clusters were subjected to KEGG pathway enrichment. Further classification and statistical analyses were carried out (Figure 4). In Cluster 1, 78 proteins were involved in metabolic pathways. Protein expression in Cluster 1 was continuously down-regulated, which indicated that the metabolic activity of the salivary glands continues to decrease during the degeneration of the salivary glands. Twenty-four proteins were involved in oxidative phosphorylation. The decreasing expression of the proteins involved in oxidative phosphorylation indicates that the energy demands of the salivary glands decrease during the salivary gland degeneration process.

Protein expression in Cluster 2 was continuously up-regulated. In Cluster 2, six proteins were involved in the necroptosis pathway. Necroptosis is a caspase-independent programmed form of necrosis or inflammatory cell death. This finding indicates that cell necrosis continues during salivary gland degeneration. These proteins play a role in promoting salivary gland degeneration during this physiological process.

Unnecessary or damaged proteins can be tagged by ubiquitin molecules. Proteasomes are protein complexes that can degrade these proteins. Some proteins in Cluster 3 are involved in proteasome formation. The expression of these proteasome-synthesis-associated proteins increased 24 h after female tick engorgement. This finding indicates that a large number of unnecessary proteins produced by the degeneration of salivary glands can be rapidly degraded in the subsequent period, allowing salivary gland degeneration to occur continuously.

### PPI Analysis of Differentially Expressed Proteins

**FIGURE 5:**
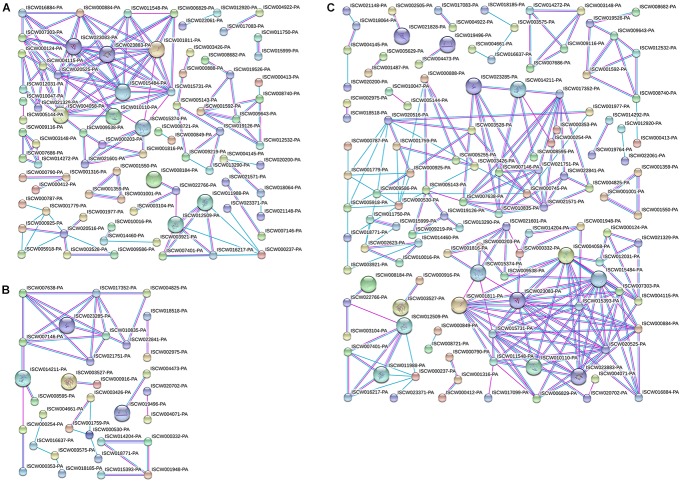
Protein interactions analyzed for differentially expressed proteins in Clusters 1 and 2. **(A)** Protein interaction analysis of differentially expressed proteins in Cluster 1. **(B)** Protein interaction analysis of differentially expressed proteins in Cluster 2. **(C)** Protein interaction analysis of differentially expressed proteins in Clusters 1 and 2.

Differentially expressed proteins in Cluster 1 (Figure 5A) and Cluster 2 (Figure 5B) were analyzed for protein interactions. In addition, all the differentially expressed proteins from both Clusters 1 and 2 were analyzed together (Figure 5C) for protein interactions. The results directly reflect the interactions between these differentially expressed proteins. This PPI analysis provides a basis for further analysis of the signaling pathways involved in salivary gland degeneration.

### RNAi Results

**FIGURE 6:**
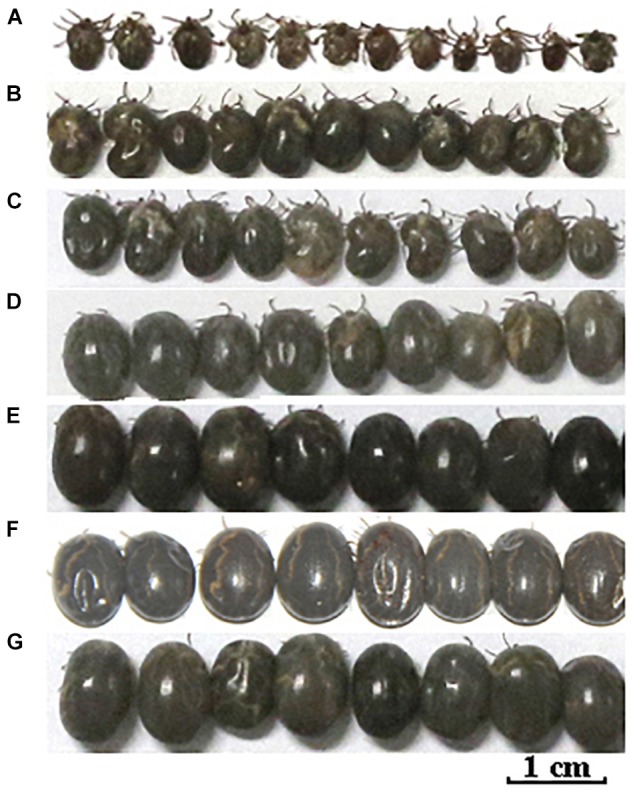
Energy metabolism and cell apoptosis related gene knockdown phenotype in female ticks injected with dsRNA. After the hungry female was injected with dsRNA, the strong ones were selected and fed to the rabbit’s ears until they were engorgement. The ticks injected with dsRNA of the GFP gene or nothing injected was used as control. **(A)** injected F0F1-type ATP synthase dsRNA; **(B)** injected Cytochrome C dsRNA; **(C)** injected NADH-ubiquinone oxidoreductase dsRNA; **(D)** injected AIF dsRNA; **(E)** injected Lysosomal acid phosphatase dsRNA; **(F)** control group, injected GFP dsRNA; **(G)** control group, nothing was injected.

**FIGURE 7:**
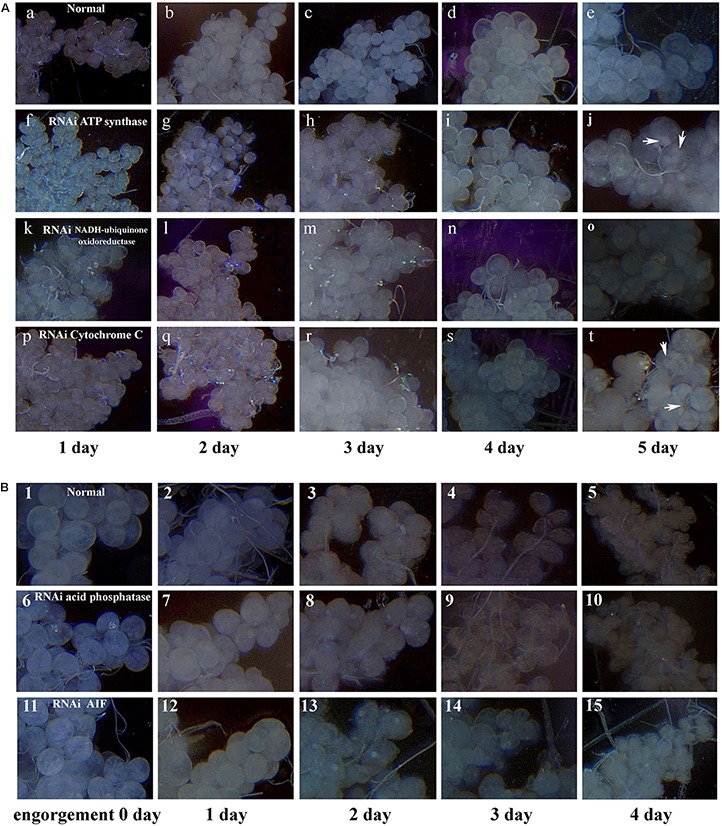
Digital micrographs of salivary glands of females *H. longicornis*. **(A)** Phenotype changes of salivary glands after RNAi in unfed females. *X*-axis shows the time after injection with dsRNA. One day after injection with dsRNA, the ticks were fed on the ears of rabbits. Arrows show sites with masses. **(a–e)** Control group; **(f–j)** injected ATP synthase dsRNA; **(k–o)** injected NADH-ubiquinone oxidoreductase dsRNA; **(p–t)** injected cytochrome C dsRNA. **(B)** Phenotype changes of salivary glands after RNAi in engorged females. **(1–5)** Control group, nothing was injected; **(6–10)** injected lysosomal acid phosphatase dsRNA; **(11–15)** injected AIF dsRNA.

After data analysis, some proteins involved in energy metabolism were selected for RNAi. The expression of F0F1-type ATP synthase, NADH-ubiquinone oxidoreductase, and cytochrome C continued to decrease during salivary gland degeneration in female ticks. This result indicates that the energy requirement of the salivary gland decreases during the process of degeneration. This finding indicates that these proteins play an important role in regulating the growth and development of salivary glands. The decreased expression of these proteins may directly hinder the development of salivary glands and can even affect whole-body development in ticks. To further examine the roles of these energy-production-related proteins in ticks, we knocked down F0F1-type ATP synthase, NADH-ubiquinone oxidoreductase, and cytochrome C using RNAi. Figure 6 shows the phenotypic changes of the engorged ticks after RNAi. Figure 7A shows the salivary gland morphological changes of the ticks after RNAi.

After F0F1-type ATP synthase, NADH-ubiquinone oxidoreductase, and cytochrome C were knocked down by RNAi, the acini of salivary glands changed significantly, and a white mass appeared in the acini. This is more prominent in salivary gland of engorged ticks after interference by F0F1-type ATP synthase (Figures 7A-j). In the meantime, the weights of the engorged female ticks decreased significantly (Table 1). The spawning capacity and hatching rates also decreased significantly compared with those of the control group (Table 1). In particular, the female ticks could not even lay eggs after F0F1-type ATP synthase was knocked down by RNAi.

**Table 1:** Physiological data for ticks after RNAi.

Proteins	Time required for engorgement (*d*) (Ave ±*SD*)	Mortality rate (%)	Engorged weight (mg) (Ave ±*SD*)	Oviposition amount (Ave ±*SD*)	Egg hatching rate (%) (Ave ±*SD*)
F0F1-type ATP synthase	8.2 ± 0.7	22.2	19.9 ± 2.9	–	–
NADH-ubiquinone oxidoreductase	6.6 ± 0.5	17.8	62.6 ± 10.1	451 ± 45	45.7 ± 7.8
Cytochrome C	6.2 ± 0.6	26.7	68.4 ± 11.3	463 ± 54	44.9 ± 9.4
AIF	5.9 ± 0.7	15.6	104.0 ± 17.8	1354 ± 38	62.1 ± 10.2
Lysosomal acid phosphatase	5.9 ± 0.8	17.8	164.1 ± 21.2	1603 ± 61	78.3 ± 8.9
GFP	5.8 ± 0.6	13.3	200.9 ± 27.1	1793 ± 59	83.3 ± 7.3
No injections	5.9 ± 0.7	15.6	204.8 ± 22.9	1879 ± 63	83.7 ± 4.6

*“–”, No eggs; Ave, average; SD, standard deviation.*

Some proteins involved in cell apoptosis were selected for RNAi. High expression of AIF and lysosomal acid phosphatase promoted apoptosis. Knock down of the two genes by RNAi showed that interference of AIF slowed down the degeneration of salivary glands of female ticks (Figure 7B). Especially, the boundary of the acinis became blurred after the tick engorged 96 h, but the shape remained round (Figure 7B-15). Acini of salivary glands in control group were obviously shrunken (Figure 7B-5), suggesting that high expression of AIF promoted the degeneration of salivary gland. However, salivary glands still degenerated after interference of lysosomal acid phosphatase, being similar to the degeneration of normal group. Interestingly, we also interfered the two genes in unfed female ticks. After AIF was knocked down, the engorged weights, oviposition amounts, and egg hatching rates of the female ticks decreased slightly. RNAi of AIF caused normal cellular physiological activity to be affected, thereby affecting the development of female ticks. However, after RNAi of lysosomal acid phosphatase, there was no significant change in the engorged weights, amount of spawning, and egg hatching rates of the female ticks. The markers of apoptosis can only reflect the situation of apoptosis. The expression of the markers cannot directly influence the apoptosis of cells, and cannot affect the degeneration of salivary gland.

To prove that the RNAs of these genes were indeed knocked down by RNAi, qPCR analysis was performed to examine the expression of these genes after RNAi. The results showed that the RNAs of these five genes were significantly silenced in both the whole-bodies of the ticks and in the salivary glands, proving that the interference technique was effective (Table 2).

**Table 2:** Gene expression silencing by RNAi.

dsRNA	Gene expression silencing (%) (Ave ±*SD*)	
	Salivary glands	Whole ticks
F0F1-type ATP synthase	47.46 ± 3.05	88.35 ± 4.71
NADH-ubiquinone oxidoreductase	93.87 ± 0.65	80.99 ± 2.09
Cytochrome C	89.83 ± 3.04	66.58 ± 17.42
AIF	76.75 ± 6.33	80.03 ± 10.6
	69.25 ± 4.11 (RNAi of engorged tick)	74.46 ± 7.23 (RNAi of engorged tick)
Lysosomal acid phosphatase	78.26 ± 16.39	46.39 ± 4.93
	71.23 ± 7.58 (RNAi of engorged tick)	71.21 ± 9.05 (RNAi of engorged tick)

*Ave, average; SD, standard deviation*.

## Discussion

Ticks are either forced to detach from the host body surface when they attain a “critical weight” after a period of blood sucking or may detach naturally after engorgement, and the salivary glands of these ticks degenerate after a certain period (Lomas et al., 1998). During salivary gland degeneration, either cell apoptosis or both apoptosis and autophagic death (Zakeri et al., 1995; Scopinho et al., 2008) can lead to many changes in protein expression. In this study, iTRAQ quantitative proteomic technology was used to study global changes in protein levels during salivary gland degeneration. iTRAQ quantitative proteomic technology has received wide attention since its introduction. This technology is often used in the research of animals with different classification levels (Gao et al., 2018; Jia et al., 2018; Song et al., 2018). However, the use of iTRAQ quantitative proteomics to study these important parasitic organisms—ticks have rarely been reported. Only a few articles have reported the use of iTRAQ technology for tick-related research, including studies on the spread of disease (Villar et al., 2012), co-evolution of symbiotic bacteria (Cotté et al., 2014) and host immunity (Kongsuwan et al., 2010). To the best of our knowledge, this is the first time that quantitative proteomics has been used to study changes in protein levels that occur during salivary gland degeneration in ticks. In-depth studies of the specific programmed cell death that occurs in the salivary glands of ticks has great significance for the control of ticks and for indirect prevention of tick disease.

For further analysis, we clustered the proteins according to expression patterns. Eventually, the proteins that exhibited a 1.5-fold change in expression were divided into four clusters. Opposite trends for changes in protein expression levels between Clusters 1 and 2 were observed from the day of female engorgement (0 h) to the 3rd day post engorgement (72 h). Although there were some slight fluctuations or the change in the expression of a protein between the two post-engorgement stages was not 1.5-fold, the overall trend for Cluster 2 was up-regulation. In contrast, the overall trend for proteins in Cluster 1 was down-regulation. The main functional categories of proteins in Cluster 2 were as follows: (1) proteins related to apoptosis, such as AIF; (2) apoptotic markers, such as lysosomal acid phosphatase; (3) enzymes involved in the degradation of chromosomal DNA and proteins in cells, such as DNA damage-inducible protein, DNA damage repair/toleration protein, serine carboxypeptidase, and aspartic protease; (4) proteins associated with cell structure and components, such as histone; (5) proteins with multiple cellular functions, such as splicing factor, which is involved in the splicing of RNA precursors (Jangi and Sharp, 2014); and (6) proteins with unknown function.

### Cluster 2

Our results showed that the expression level of AIF increased continuously after female engorgement. Known to be involved in caspase-independent apoptosis, AIF is a mitochondrial protein that is released into the cytosol and promotes apoptosis (Candé et al., 2002). When cells are stimulated by apoptosis, AIF is released into the cytoplasm from the mitochondrial intermembrane space and enters the nucleus due to the presence of the AIF nuclear localization sequence. Then, this protein causes intracellular chromatin condensation and DNA fragmentation (Susin et al., 1999). To the best of our knowledge, while AIF has been found in some other arthropods (Joza et al., 2008; Anitha et al., 2014), there have been no reports on AIF in ticks. The continuous high expression of AIF may be one of the main influencing factors that promotes rapid degeneration of the salivary glands of engorged female ticks. Our results also demonstrated this. When the expression of AIF was reduced by RNAi, the cells could not undergo normal apoptosis, thus affecting the normal cell cycle, which affected all the physiological functions of the ticks.

Acid phosphatase activity can reflect the cell histolysis status. Acid phosphatase activity is inversely proportional to ATPase activity and membrane integrity (Furquim et al., 2008a). This study found that the lysosomal acid phosphatase content was lowest in the salivary glands at the time of engorgement (0 h), then increased gradually over time, and maximum expression was attained at 72 h post engorgement, which could be a result of the occurrence of apoptosis or autophagy in the tick salivary gland cells, leading to the integrity of the cell membrane being disrupted and in turn to a decrease in ATP production. Simultaneously, in contrast to the increase in acid phosphatase activity, ATPase activity slowly decreased (Cluster 1) (Nunes et al., 2006; Scopinho et al., 2008). In conclusion, acid phosphatase appears to be an indicator of the extent of cell histolysis in tick salivary glands. However, when acid phosphatase was knocked down by RNAi, the ticks were not affected much. This is an observation that should be considered in future research.

After engorgement, the cells of the female tick salivary glands undergo programmed cell death. The degraded salivary gland cells exhibit autophagic vacuoles (Harris and Kaufman, 1985), blebbing (Furquim et al., 2008b), chromatin condensation (Fawcett et al., 1982; Scopinho et al., 2008), and chromosomal DNA damage (Freitas et al., 2007; Galluzzi et al., 2012). Experimental data from this study show that some proteins associated with DNA fragmentation and protein degradation continue to be highly expressed after tick engorgement. DNA damage-inducible proteins play an important role in DNA damage repair, protein trafficking, and the cell cycle (Voloshin and Camerini-Otero, 2007; Ren et al., 2009; Trempe et al., 2016). The ATP-dependent DNA helicase II has multiple functions in DNA metabolism, including DNA replication, repair, and recombination (Cooper et al., 2015). The ku P80 DNA helicase also functions in DNA break repair (Koike et al., 1996). Because these enzymes have the ability to repair DNA damage, increased expression of these enzymes indicates that the salivary gland cells accelerate DNA fragmentation at this stage, thus causing the cells to attempt to repair the damaged DNA. Simultaneously, a large amount of protein begins to be degraded. Rapid degradation of a large amount of protein requires many 26S complexes from the ubiquitin-proteasome pathway. The degradation of proteins is associated with ubiquitin-activating enzyme (E1), ubiquitin-conjugating enzyme (E2), and ubiquitin ligase (E3) and requires the participation of the 26S proteasome (Hershko and Ciechanover, 1998). The up-regulation of ubiquitin ligase (E3) and expression of the 26S proteasome regulatory subunit observed in this study may indicate an increase in expression of the ubiquitin-proteasome system, which catalyzes a great majority of protein degradation (Zhao et al., 2015; Collins and Goldberg, 2017). Serine carboxypeptidase and aspartic protease play different roles in protein degradation. The continuous high expression of these proteins reflects the rapid decomposition of various components in cells during programmed cell death.

### Cluster 1

Overall, the proteins in Cluster 1 were down-regulated from the day of female engorgement (0 h) to the 3rd day post engorgement (72 h). These proteins were mostly enzymes associated with the mitochondrial respiratory chain and some enzymes associated with carbohydrate and lipid metabolism. The mitochondrial respiratory chain can provide energy to cells and tissues. Mitochondria are the main sites of cellular energy metabolism. In addition, mitochondria can control cell proliferation (Rustin, 2002) and apoptosis (Kroemer and Reed, 2000; Wang, 2001). The salivary glands degenerated rapidly after *H. longicornis* engorgement, and the energy needs of the cell were greatly reduced; the energy was provided by the respiratory chain. This study found that the expression levels of some proteins associated with the respiratory chain and energy production, such as NADH: ubiquinone oxidoreductase, cytochrome C, cytochrome C oxidase, glycerol-3-phosphate dehydrogenase, and ATP synthase, decreased during this period. NADH: ubiquinone oxidoreductase can catalyse the reversible redox conversion of ubiquinone and NADH to ubiquinol and NAD^+^, respectively (Brandt, 2006), which is an important part of the respiratory chain. Cytochrome C and cytochrome C oxidase are important components of the respiratory chain and play important roles in redox and energy metabolism (Fernández-Vizarra et al., 2009). Once the levels of these two proteins decrease, the mitochondria loses part of its functionality, which can lead to necrosis due to ATP depletion. Similarly, the glycerol-3-phosphate dehydrogenase on the inner mitochondrial membranes of eukaryotes can channel electrons into the respiratory chain and is also a major donor of electrons to the electron transport chain (Yeh et al., 2008). The decrease in the expression of these proteins directly reflects a reduction in cellular energy requirements. The levels of these enzymes also decrease when mitochondria are destroyed. ATP synthase is an enzyme that generates ATP. Reduction in the amount of ATP synthase expression directly causes a reduction in ATP synthesis. ATP is the most direct source of energy in organisms and is the most commonly used “energy currency.” In addition, ATP can trigger many different cell responses, and low levels of ATP promote cell death (Halestrap, 2005). This study found that the expression level of ATP synthase decreased from the day of female engorgement (0 h) to 48 h post engorgement; the ATP synthase level at 72 h was basically equal to that at 48 h. After 48 h, the ATP synthase level no longer decreased, and from that point on, the amount of ATP present was probably the minimum amount required for maintaining the lowest level of physiological activity of the cell.

Cluster 1 also contained some enzymes associated with carbohydrate and lipid metabolism, such as UTP-glucose-1-phosphate uridylyltransferase, which plays a central role in carbohydrate metabolism. This enzyme is essential for glycogenesis and plays an important role in glycogen synthesis, glycoprotein synthesis, and glycolipid synthesis (Sandhoff et al., 1992; Alonso et al., 1995). 3-Hydroxyacyl-CoA dehydrogenase mainly participates fatty acid metabolism and can catalyze the penultimate reaction in β-oxidation of fatty acids (Heslegrave et al., 2012). Enoyl-CoA hydratase also plays an important role in fatty acid metabolism and can catalyze the second step of β-oxidation in fatty acid metabolism (Bahnson et al., 2002). The decrease in expression of these enzymes directly reflects the gradual decrease in energy production and metabolic activity in the cells during the process of salivary gland degeneration.

The expression of some proteins associated with signal transduction also continued to be down-regulated, such as protein tyrosine phosphatase and Ca^2+^ sensors. Protein tyrosine phosphatase can remove phosphate groups from phosphorylated tyrosine residues that confer a variety of specific biological functions to these proteins. This enzymes is a key regulatory component of signal transduction pathways (such as the MAP kinase pathway) and cell cycle regulation processes, including cell growth, proliferation, differentiation, and transformation (Denu and Dixon, 1998; Paul and Lombroso, 2003; Zhang et al., 2010). In the signal transduction pathway mediated by G-protein-coupled receptors, Ca^2+^ sensors can bind intracellular Ca^2+^ to form a calcium-sensing protein complex and can then control a series of physiological processes (Burgoyne and Weiss, 2001). Because these two proteins play important roles in the regulation of many types of cellular physiological activities, decreased expression of these proteins can directly reflect a decrease in cell vitality. The solute carrier family 12 (SLC12) is composed of nine genes that encode chloride co-transporters. These proteins are distributed in the plasma membrane and are mainly used for the exchange of Na^+^ and/or K^+^ ions and Cl^-^ ions (Bazúa-Valenti et al., 2016). The continuous decrease in the expression of this enzyme may indicate that the ion exchange rate decreased during this period.

The members of the caspase family are mostly apoptosis promoters or effectors and play important roles in cell apoptosis. Caspases can act on different substrates to cause cell apoptosis. The cells exhibited some morphological changes and biochemical changes upon apoptosis, including cell membrane foaming, apoptotic vesicle formation, detachment from the extracellular matrix, formation of pyknotic nuclei, phospholipid exposure, and DNA fragmentation (Yuan et al., 2016). The only caspase family proteins from ticks listed in NCBI GenBank are caspase-1, -2, -3, and -8. This study found that the caspase-1 protein was down-regulated. Yu et al. (2017) showed that the expression of caspase-1 increased before female engorgement (after attachment to the host 5–7 days), so it is possible that the expression of this protein started to decrease after engorgement. We determined that caspase was produced in the early stages of salivary gland cell apoptosis, and the expression of the caspase protein decreased significantly after engorgement.

Excessive copper ions in the body lead to the production of reactive oxygen species (ROS), which can cause cellular damage and cell death (Landolfo et al., 2008; Hegde et al., 2011). The copper chaperone is a type of superoxide dismutase (SOD). SOD has important antioxidant activities, and a decrease in SOD levels can lead to massive oxidative stress and to cell death (Ott et al., 2007).

### Clusters 3 and 4

The expression of proteins classified into Clusters 3 and 4 was irregular among the different post-engorgement time points. The expression of the proteins in Cluster 3 increased from 0 to 24 h post female engorgement, after which the expression remained stable and exhibited little change. The main difference between the protein expression characteristics of Clusters 4 and 3 was that the proteins in Cluster 4 were down-regulated from 0 to 24 h post engorgement, and the expression of these proteins was not stable over the next 48 h. There were many types of proteins in Clusters 3 and 4, exhibiting a variety of functions, including proteins associated with lipid, cholesterol, and purine nucleobase metabolism; proteins associated with transcriptional regulation, splicing, translation, and folding; proteins involved in the formation of the cytoskeleton; and proteins associated with signal transduction. Due to the complexity of cellular metabolic activity, protein expression levels are dynamic and constantly changing. The expression levels measured for these proteins during the long post-tick-bite interval were not stable. In the future, these proteins should be further studied using shorter post-tick-bite time intervals.

## Ethics Statement

All experimental procedures were approved by the Animal Ethics Committee of Hebei Normal University (protocol number: IACUC-157026). Because the ticks in this study needed blood to grow, they involved feeding with animal blood. We didn’t kill the animals, just let the ticks suck a little blood. This experiment conformed to the animal ethics regulations proposed by the animal protection organization.

## Author Contributions

HW designed experiments, analyzed the data, performed the experiments, and wrote the initial manuscript. XZ, XW, BZ, MW, XH, and RW performed the experiments. XY edited the manuscript. SR and YH prepared the figures. JL designed the experiments and correction of the manuscript.

## Conflict of Interest Statement

The authors declare that the research was conducted in the absence of any commercial or financial relationships that could be construed as a potential conflict of interest.

## Footnotes

1 http://www.pantherdb.org/

2 http://www.kegg.jp/kegg/

3 http://string-db.org/

4 http://www.pantherdb.org/

## Supplementary Material

FIGURE S1 Saturation curve of transcriptome sequencing data. The horizontal axis shows the number of reads (M), and the vertical axis shows the number of genes (K). An RPKM of 0.1 was set as the threshold for gene expression.

FIGURE S2 Unigene length distribution map. The abscissa represents the different length intervals of the unigenes; the ordinate indicates the number of unigenes in certain length intervals.

FIGURE S3 Gene Ontology (GO) function annotations for all the differentially expressed proteins.

FIGURE S4 Gene Ontology functional annotations of differentially expressed proteins in Cluster 1. The percentages of proteins assigned to the different terms are shown. **(A)** Molecular function; **(B)** biological process; **(C)** cellular component.

FIGURE S5 Gene Ontology functional annotations of differentially expressed proteins in Cluster 2. The percentages of proteins assigned to the different terms are shown. **(A)** Molecular function; **(B)** biological process; **(C)** cellular component.

FIGURE S6 Gene Ontology functional annotations of differentially expressed proteins in Cluster 3. The percentages of proteins assigned to the different terms are shown. **(A)** Molecular function; **(B)** biological process; **(C)** cellular component.

FIGURE S7 Gene Ontology functional annotations of differentially expressed proteins in Cluster 4. The percentages of proteins assigned to the different terms are shown. **(A)** Molecular function; **(B)** biological process; **(C)** cellular component.

FIGURE S8 Protein statistics based on GO functional annotations of differentially expressed proteins.

TABLE S1 Statistics of the assembly results from Illumina sequencing.

TABLE S2 The common proteins were identified in all three experiments. Up-regulated or down-regulated proteins were also counted and recorded in this excel.
