# Supplementary figures and images for: Comprehensive Analysis of the Global Protein Changes That Occur During Salivary Gland Degeneration in Female Ixodid Ticks Haemaphysalis longicornis
Source: Front Physiol. 2019 Jan 22;9:1943. doi: 10.3389/fphys.2018.01943 (PMC6349780; doi:10.3389/fphys.2018.01943)

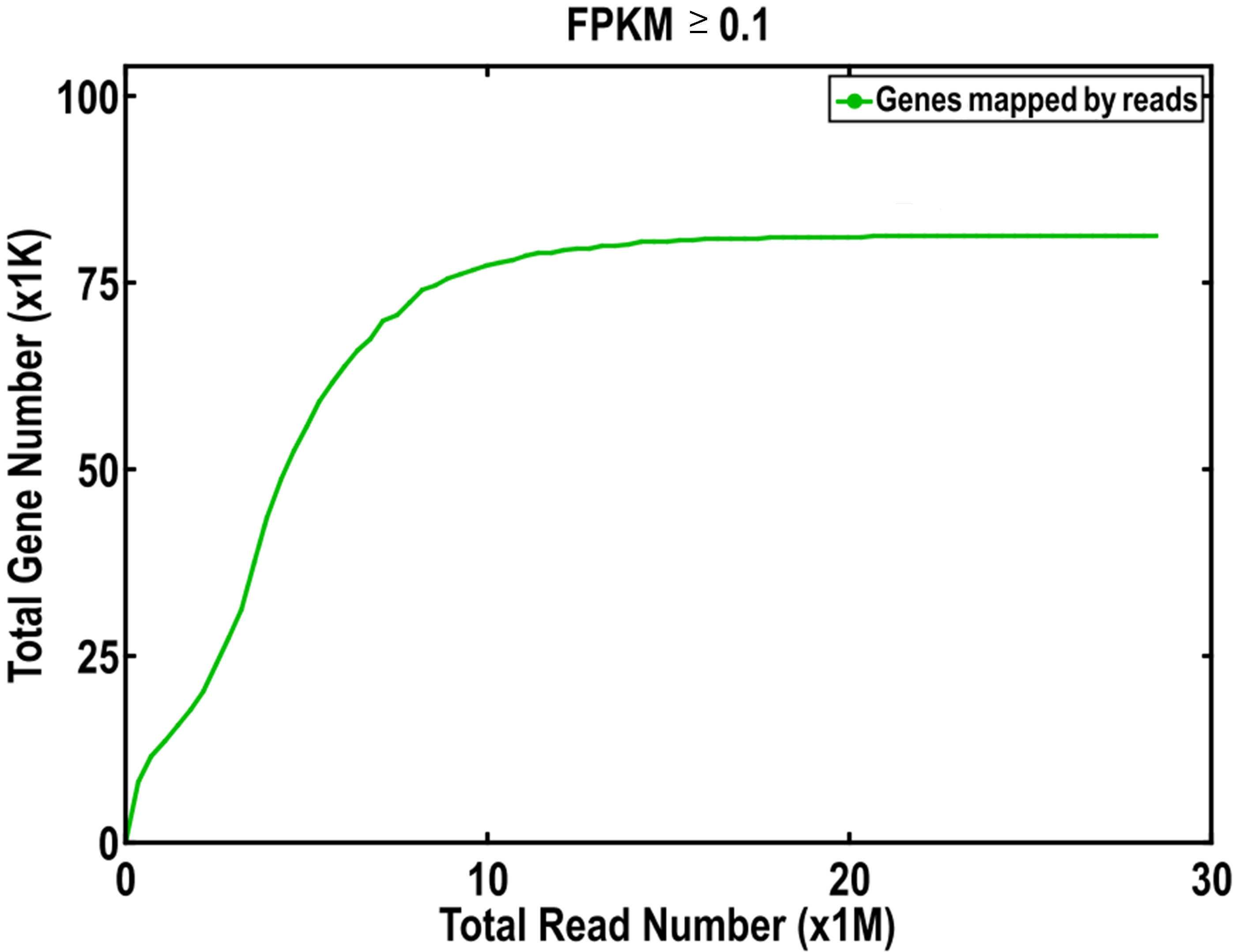

Supplement: FIGURE S1 — Saturation curve of transcriptome sequencing data. The horizontal axis shows the number of reads (M), and the vertical axis shows the number of genes (K). An RPKM of 0.1 was set as the threshold for gene expression. [file Image_1.JPEG]

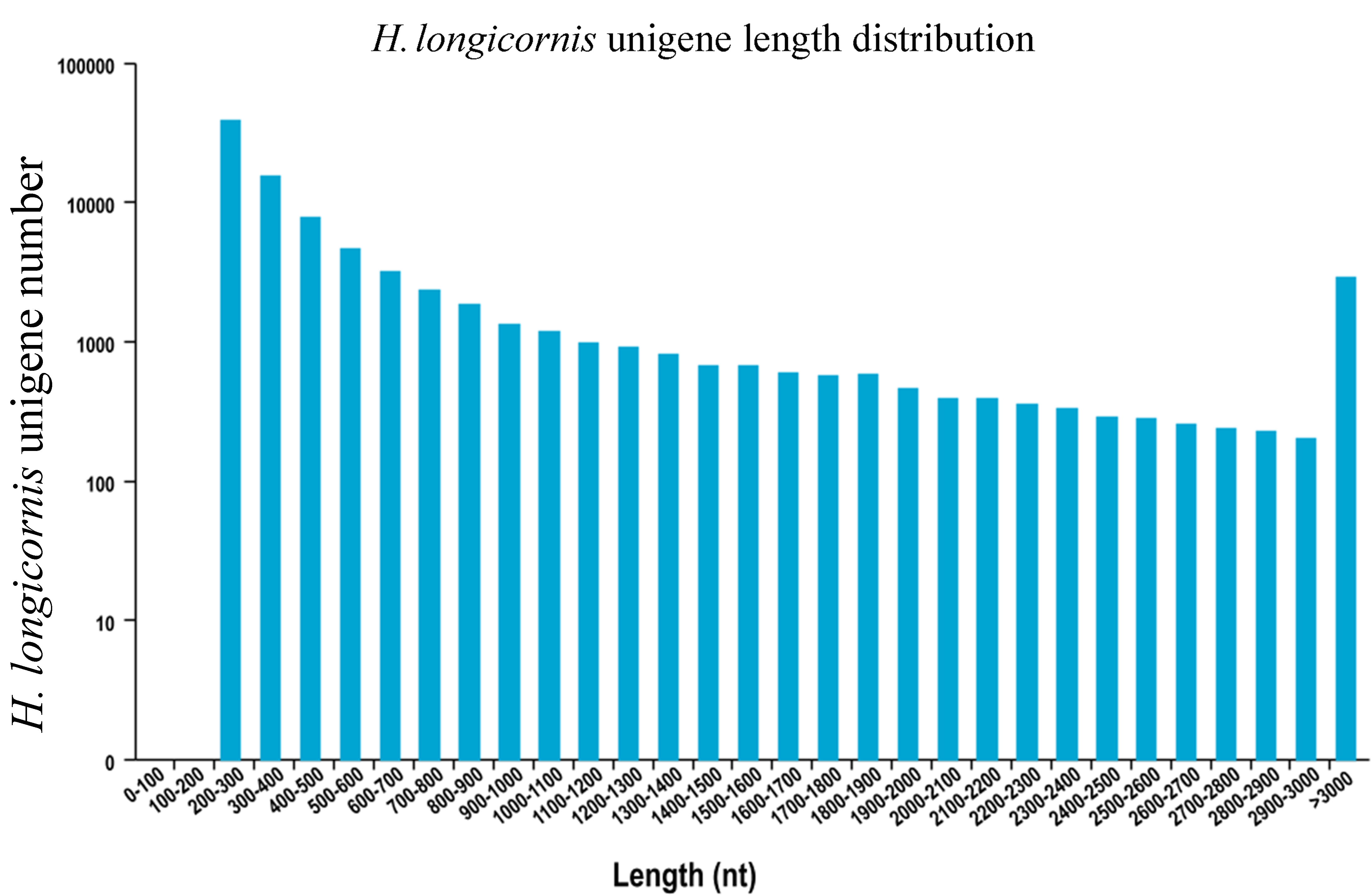

Supplement: FIGURE S2 — Unigene length distribution map. The abscissa represents the different length intervals of the unigenes; the ordinate indicates the number of unigenes in certain length intervals. [file Image_2.JPEG]

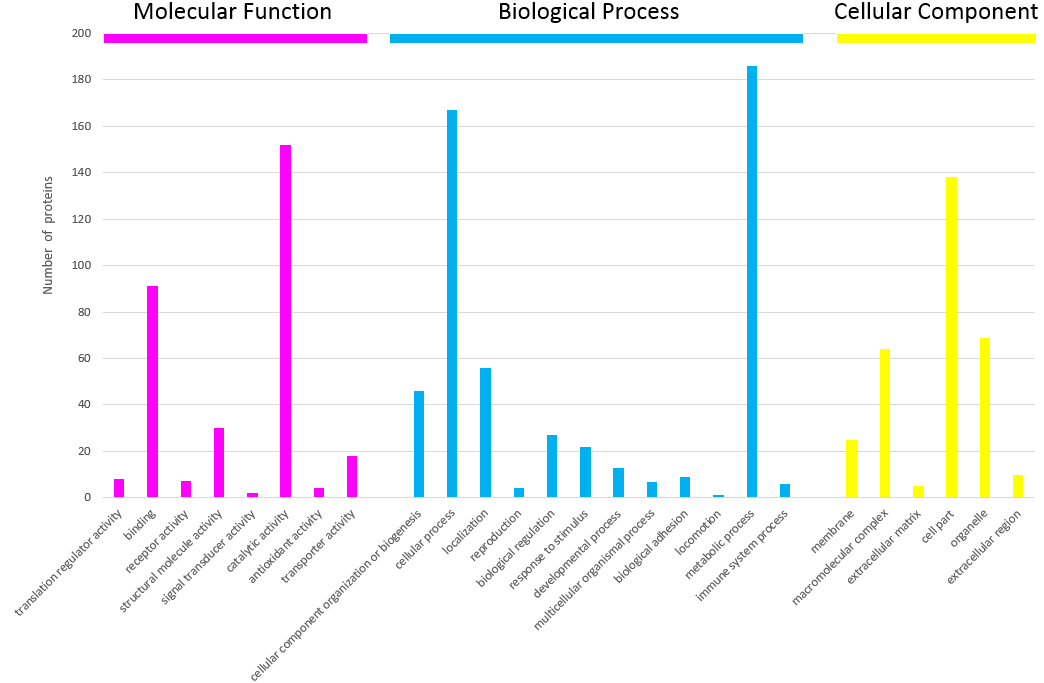

Supplement: FIGURE S3 — Gene Ontology (GO) function annotations for all the differentially expressed proteins. [file Image_3.JPEG]

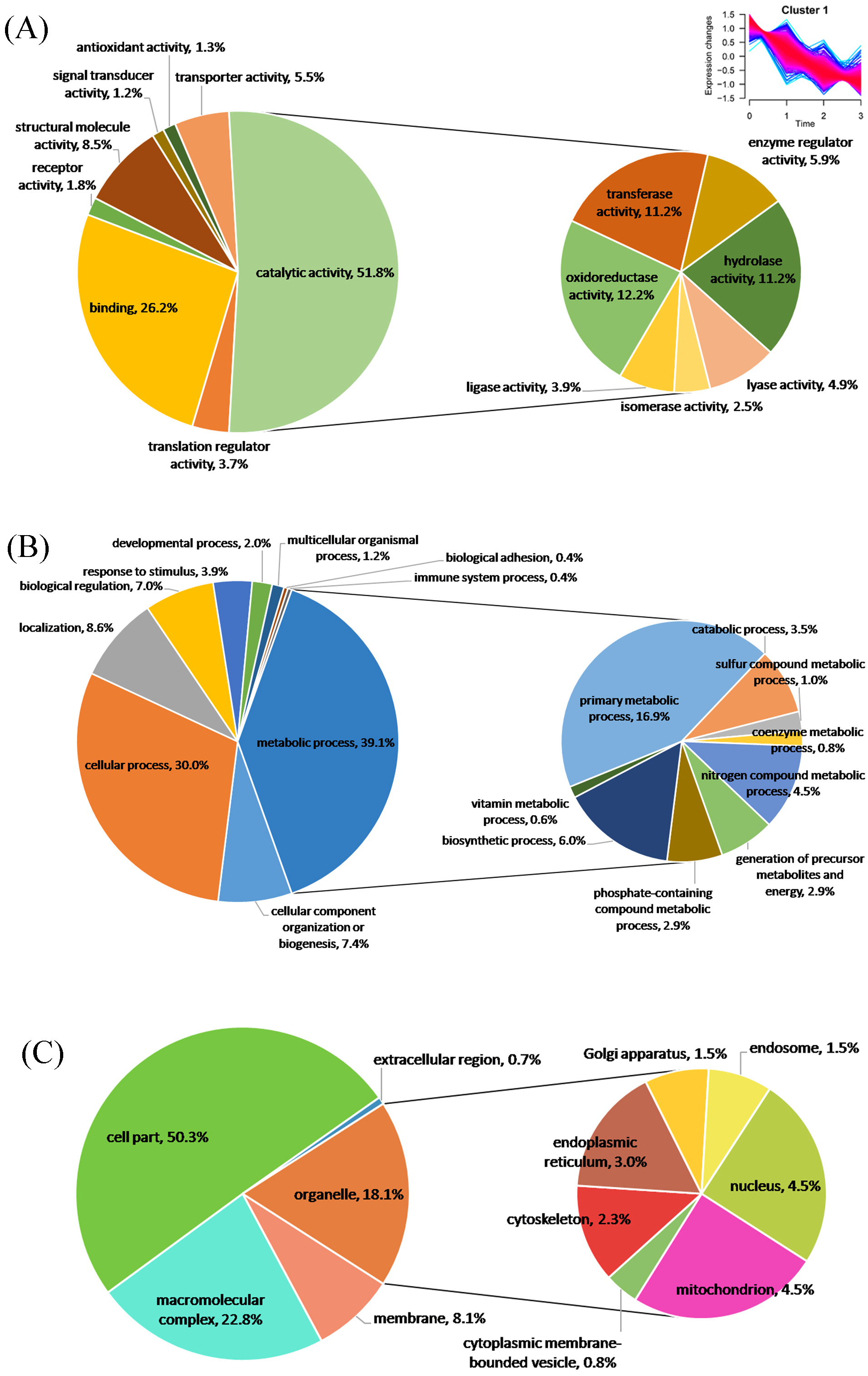

Supplement: FIGURE S4 — Gene Ontology functional annotations of differentially expressed proteins in Cluster 1. The percentages of proteins assigned to the different terms are shown. (A) Molecular function; (B) biological process; (C) cellular component. [file Image_4.JPEG]

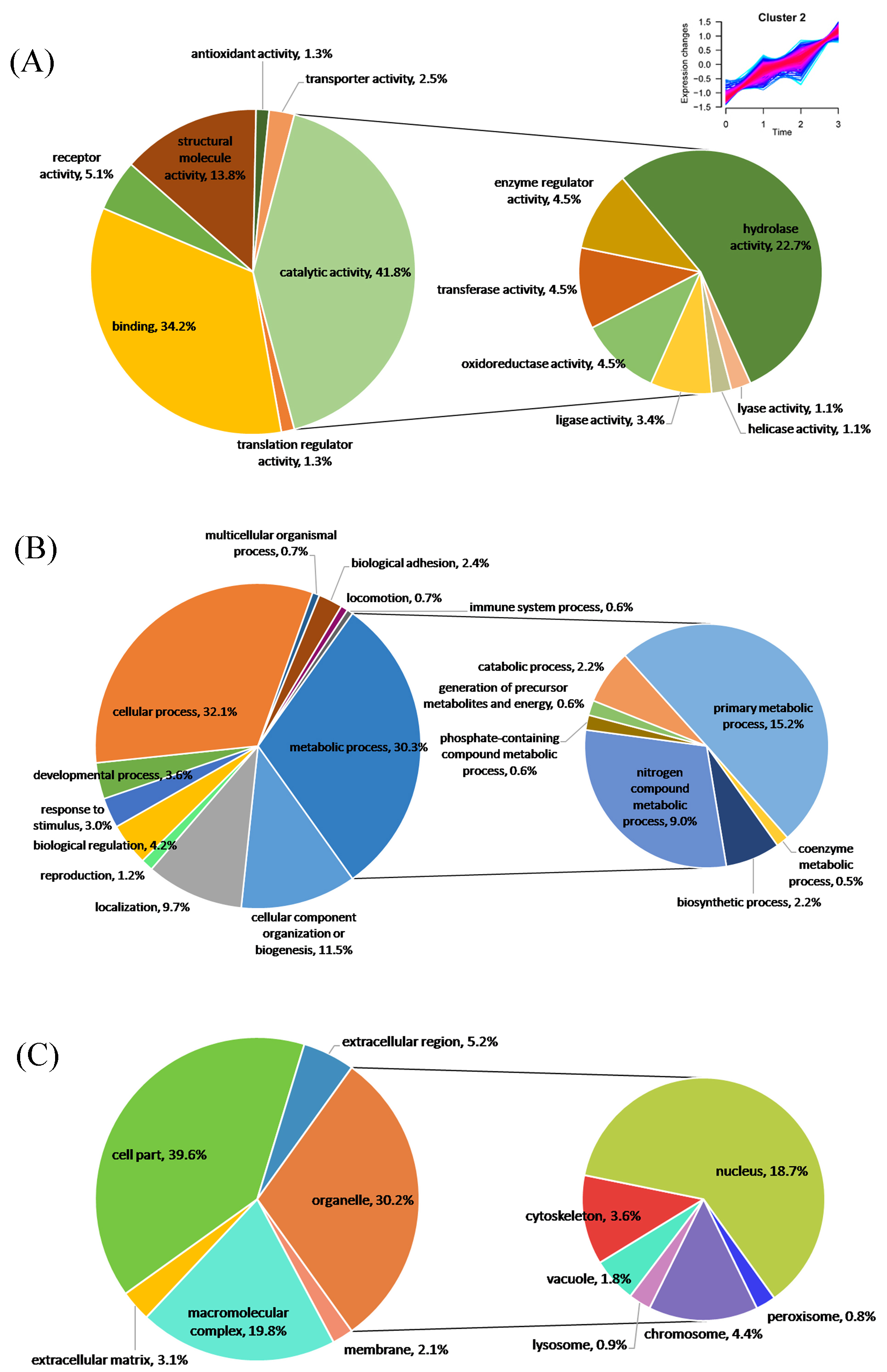

Supplement: FIGURE S5 — Gene Ontology functional annotations of differentially expressed proteins in Cluster 2. The percentages of proteins assigned to the different terms are shown. (A) Molecular function; (B) biological process; (C) cellular component. [file Image_5.JPEG]

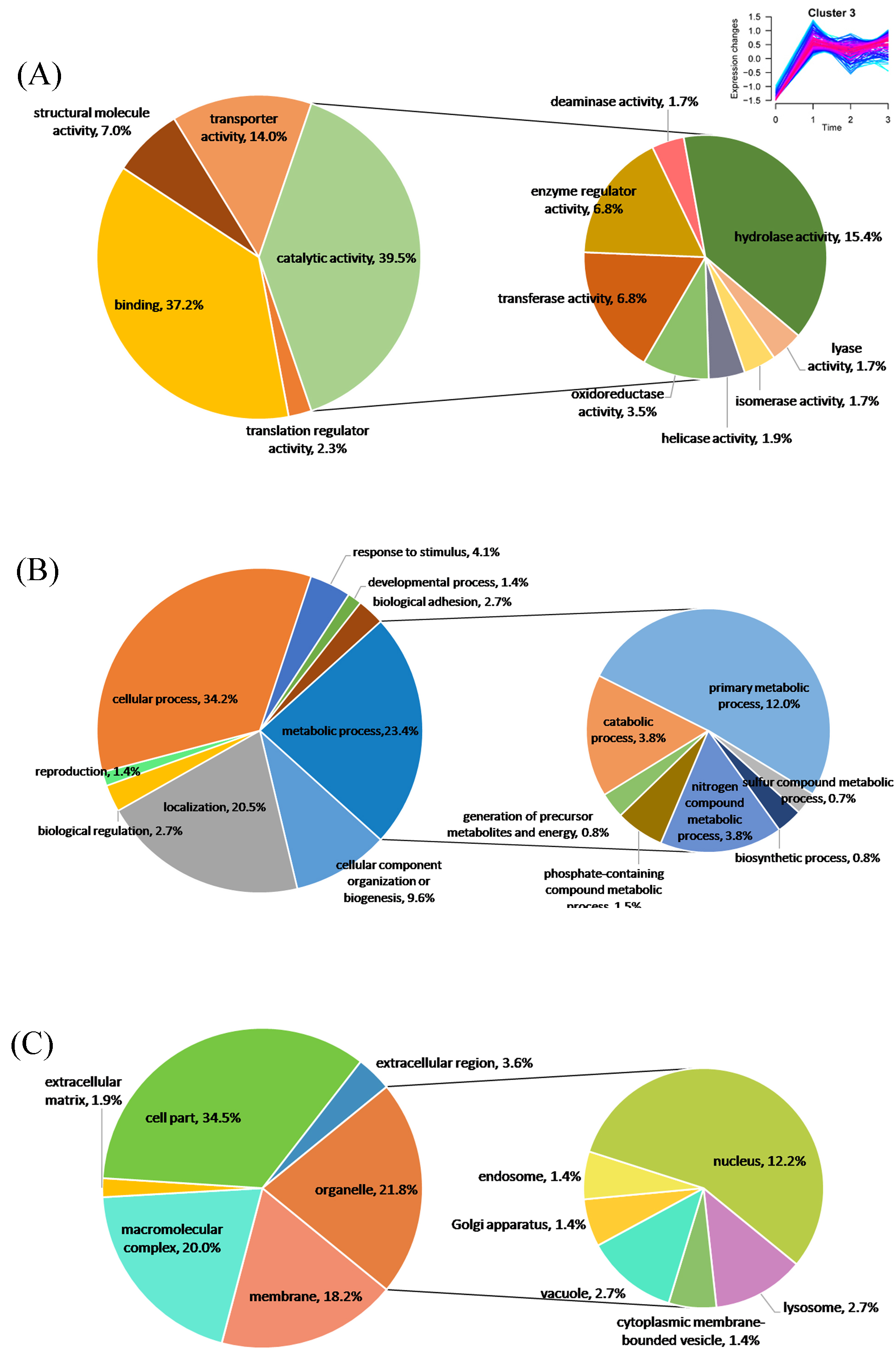

Supplement: FIGURE S6 — Gene Ontology functional annotations of differentially expressed proteins in Cluster 3. The percentages of proteins assigned to the different terms are shown. (A) Molecular function; (B) biological process; (C) cellular component. [file Image_6.JPEG]

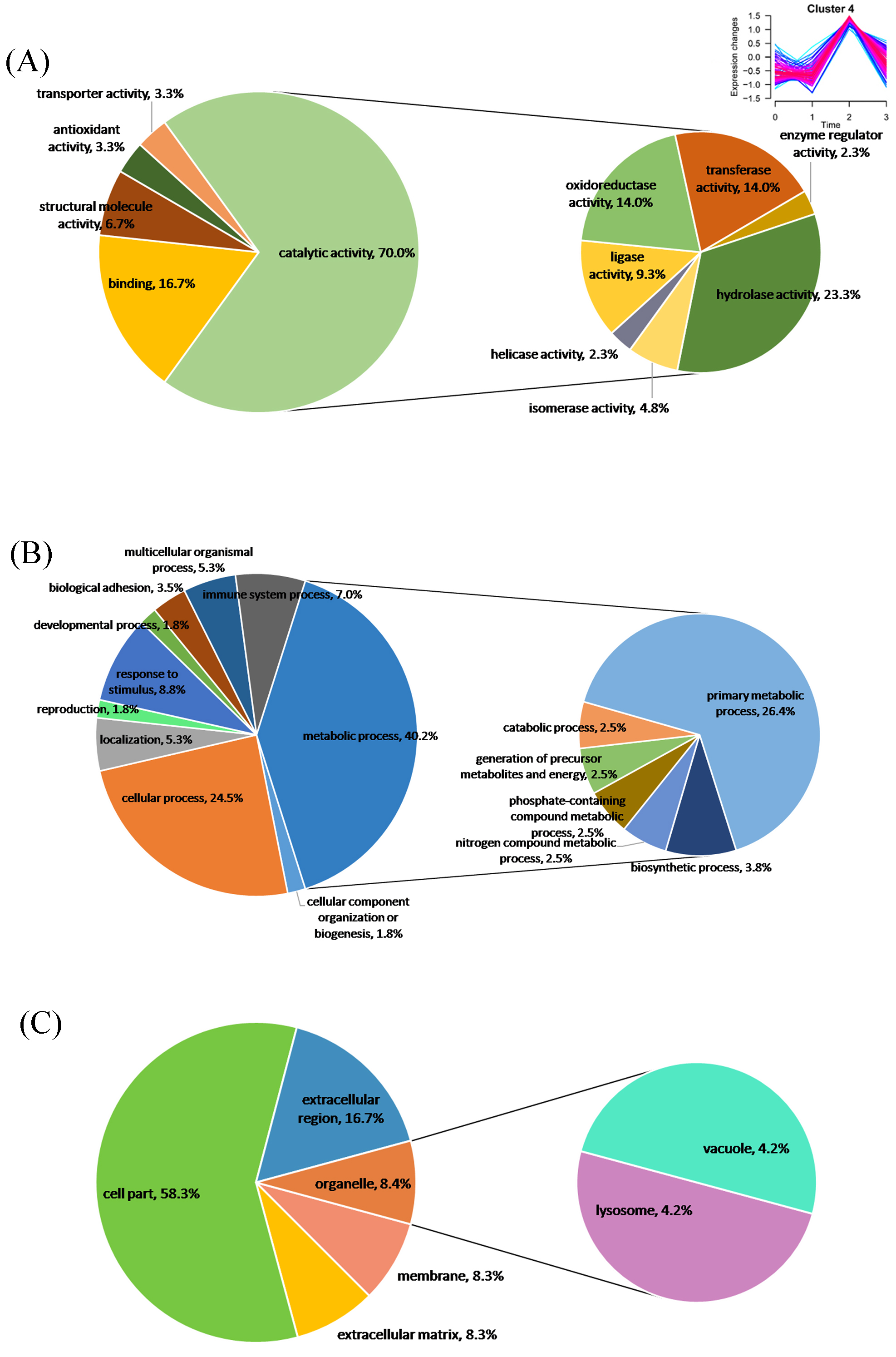

Supplement: FIGURE S7 — Gene Ontology functional annotations of differentially expressed proteins in Cluster 4. The percentages of proteins assigned to the different terms are shown. (A) Molecular function; (B) biological process; (C) cellular component. [file Image_7.JPEG]

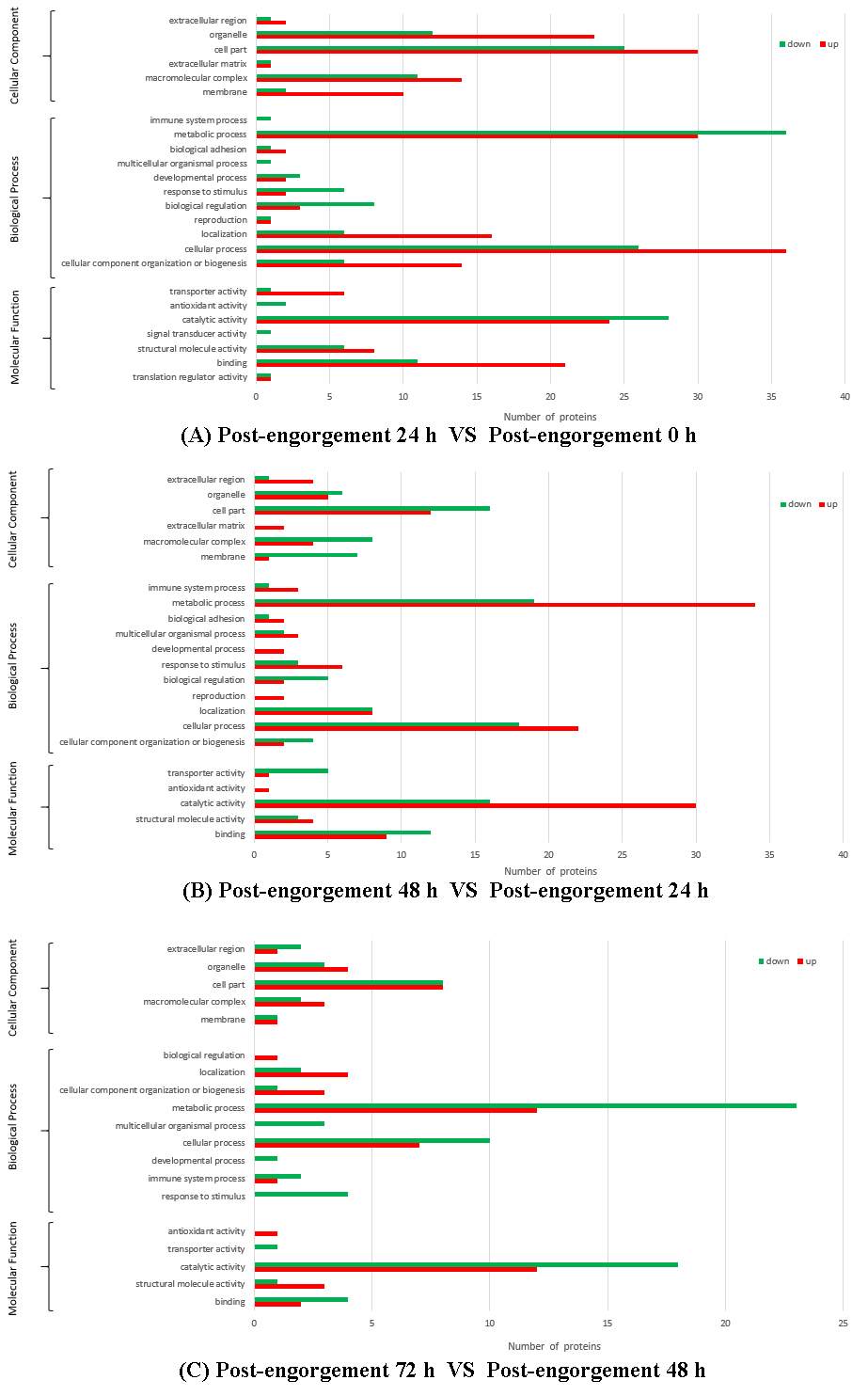

Supplement: FIGURE S8 — Protein statistics based on GO functional annotations of differentially expressed proteins. [file Image_8.JPEG]
