# Supplementary material for: Comprehensive Analysis of the Global Protein Changes That Occur During Salivary Gland Degeneration in Female Ixodid Ticks Haemaphysalis longicornis
Source: Front Physiol. 2019 Jan 22;9:1943. doi: 10.3389/fphys.2018.01943 (PMC6349780; doi:10.3389/fphys.2018.01943)
Supplement: TABLE S1 — Statistics of the assembly results from Illumina sequencing. [file Table_1.DOCX]

**Supplemental Table 1 Statistics of the assembly results from Illumina sequencing**

| Length range | Contig number | Transcript number | Unigene number |
| --- | --- | --- | --- |
| 200-300 | 2,426,888 (97.82%) | 43,444 (35.65%) | 38,325 (43.64%) |
| 300-500 | 25,672 (1.03%) | 28,556 (23.43%) | 23,012 (26.20%) |
| 500-1000 | 14,580 (0.59%) | 21,008 (17.24%) | 13,266 (15.11%) |
| 1000-2000 | 7,536 (0.30%) | 15,441 (12.67%) | 7,414 (8.44%) |
| 2000+ | 6,327 (0.26%) | 13,406 (11.00%) | 5,808 (6.61%) |
| Total number | 2,481,003 | 121,855 | 87,825 |
| Total length | 186,899,259 | 104,269,418 | 57,416,590 |
| N_50_ length | 74 | 1,716 | 1,140 |
| Mean length | 75.33 | 855.68 | 653.76 |

Length range: the contig/transcript/unigene length range; the numbers in the table indicate the number of contigs/transcripts/unigenes in the corresponding interval, and the percentages in brackets represent the proportion of contigs/transcripts/unigenes in the corresponding length range. Total number: the total number of contigs/transcripts/unigenes assembled. Total length: the total length of the contig/transcript/unigene assembly. N_50_ length: the N_50_ length of the contigs/transcripts/unigenes. Mean length: the average length of the contigs/transcripts/unigenes.
